# Visual attention modulates the asymmetric influence of each cerebral hemisphere on spatial perception

**DOI:** 10.1038/srep19190

**Published:** 2016-01-13

**Authors:** Meijian Wang, Xiuhai Wang, Lingyan Xue, Dan Huang, Yao Chen

**Affiliations:** 1 School of Biomedical Engineering, Shanghai Jiao Tong University, Shanghai, 200240, China; 2 Medical College, Qingdao University, Qingdao, 266021, China; 3 School of Quality and Technical Supervision, Hebei University, Baoding, 071002, China

## Abstract

Although the allocation of brain functions across the two cerebral hemispheres has aroused public interest over the past century, asymmetric interhemispheric cooperation under attentional modulation has been scarcely investigated. An example of interhemispheric cooperation is visual spatial perception. During this process, visual information from each hemisphere is integrated because each half of the visual field predominantly projects to the contralateral visual cortex. Both egocentric and allocentric coordinates can be employed for visual spatial representation, but they activate different areas in primate cerebral hemispheres. Recent studies have determined that egocentric representation affects the reaction time of allocentric perception; furthermore, this influence is asymmetric between the two visual hemifields. The egocentric-allocentric incompatibility effect and its asymmetry between the two hemispheres can produce this phenomenon. Using an allocentric position judgment task, we found that this incompatibility effect was reduced, and its asymmetry was eliminated on an attentional task rather than a neutral task. Visual attention might activate cortical areas that process conflicting information, such as the anterior cingulate cortex, and balance the asymmetry between the two hemispheres. Attention may enhance and balance this interhemispheric cooperation because this imbalance may also be caused by the asymmetric cooperation of each hemisphere in spatial perception.

The separate contributions of the cerebral hemispheres to various brain functions have been investigated in split-brain patients for the past century^1^, whereas the interaction between hemispheres in normal humans is far from understood. Visual spatial perception is highly dependent on interhemispheric communication. Our visual field is divided into two hemifields by its vertical meridian, and information from each half is predominantly processed by the contralateral visual cortex. However, each visual hemifield is not isolated from the other in subsequent spatial coding processes, especially with respect to processes of visuomotor integration. Evidence from monkey electrophysiological^2^,^3^ and human neuroimaging studies^4^ suggests that certain higher level cortical areas, such as the lateral intraparietal cortex (LIP) and the supplementary eye field (SEF) of the macaque monkey, are involved in processing ipsilateral information. According to previous studies, the cerebral representation of visual space can be described using two coordinate systems, namely the observer-centered (egocentric) or object-centered (allocentric) coordinates^5^. Performing visuospatial tasks, such as completing a jigsaw puzzle, involves taking into account where an object is relative to the viewer and to other objects. Representations of visual information originating from the retina are primarily defined in an egocentric reference frame in vertebrates, such as cats^6^ and humans^7^. Cerebral representations of allocentric reference frames are removed from the earliest visual information processing steps, as demonstrated in monkeys^3^ and humans^8^ (Fig. 1A). Encoding for the two spatial frames may share some brain areas^9^, and these areas may mutually influence each other^10^,^11^,^12^.

In 1935, Roelofs first described the influence of allocentric representation on egocentric spatial perception. This phenomenon was termed the “Roelofs effect”. For example, a large frame displayed to the left of an observer’s sagittal center line will make the observer perceive a target inside the frame farther to the right than its actual position. This effect was confirmed by later studies^12^. Recently, Zhou *et al.* showed the reverse effect, i.e., the influence of egocentric representation on allocentric spatial perception. These authors showed that the reaction time (RT) of allocentric position judgment was slower when allocentric and egocentric information conflicted (incompatible condition) as opposed to when allocentric and egocentric information agreed (compatible condition)^11^. This poor performance in the incompatible condition is similar to the “Simon effect”^13^ and “Eriksen flanker effect”^14^, in which conflicting task-irrelevant information affects the perception of target information.

Another interesting finding from the study by Zhou *et al.* is the asymmetry between the left and right visual hemifields while performing the incompatible task^11^. There might be several possible mechanisms behind this phenomenon. In 1995, Olson *et al.* found that certain SEF neurons in macaques encode the direction of saccades relative to an object-centered frame regardless of the egocentric coordinates^3^. These neurons are sensitive to the object-centered direction of saccades and need more interhemispheric cooperation under an incompatible condition, such as allocentric left and egocentric right or allocentric right and egocentric left (Fig. 1B,D). Moreover, brain lesions in many visual hemifield-neglect patients were reported to affect their perception on not only the lesion-contralateral but also the lesion-ipsilateral side of the visual field; however, these lesions always affect the lesion-contralateral side of a visible object^15^,^16^,^17^. Thus, the lateralized communication and interconnectivity between hemispheres^18^ might be responsible for this asymmetric influence of egocentric representation onto allocentric perception. In 1999, Botvinick *et al.* found that the anterior cingulate cortex (ACC) was involved in resolving conflicting information^19^. Functional imbalance between the left and right cortical areas involved in processing conflict, such as the ACC, may also be responsible for the asymmetry in allocentric-egocentric incompatibility. This imbalance may be due to asymmetric interhemispheric cooperation.

Few studies have investigated how the asymmetric interhemispheric cooperation related to visual spatial perception is modulated by attention^20^. Attention is a vital function that is frequently evoked to select task-relevant information from the surroundings and filter out the remaining information^21^. Endogenous visual spatial attention, one of the most important categories of visual spatial attention, can be voluntarily modulated^22^,^23^. Previous evidence suggests that the right hemisphere predominantly influences spatial attention^24^,^25^ and spatial perception^26^. In addition, spatial attention and spatial perception may share many brain circuits or pathways^9^,^27^. In our study, we investigated how visual spatial attention modulates the influence of egocentric representation on allocentric judgment.

## Methods

### Subjects

Twelve young adults (20–32 years old, 6 male, 6 female) who were students at Shanghai Jiao Tong University provided informed consent to participate in this study. All subjects had normal or corrected-to-normal vision. Seven of the participants were right eye dominant, and the remaining participants were left eye dominant according to the Miles test. The *Edinburgh Handedness Inventory*^28^ was used to assess their handedness, and all subjects were right-handed. All experimental procedures were approved by the Ethical Committee of Shanghai Jiao Tong University and conformed to the guidelines of the Declaration of Helsinki.

### Apparatus

The visual stimuli were presented on a 24-inch LCD monitor (BenQ XL2411T, 1920 × 1080 pixels, 100 Hz refresh rate) positioned 57 cm from the subject. The head position of the subject was held in place using a chin rest, and their eye position was monitored using an infrared imaging-based eye tracker (ViewPoint EyeTracker, Arrington Research). MATLAB (MathWorks) with Psychtoolbox was used to control stimulus presentation and collect manual reaction time (RT) data. The data were analyzed using Statistical Package for Social Sciences (SPSS, Inc.) and OriginPro software (OriginLab Corporation).

### Behavioral task

**Figure 1:**
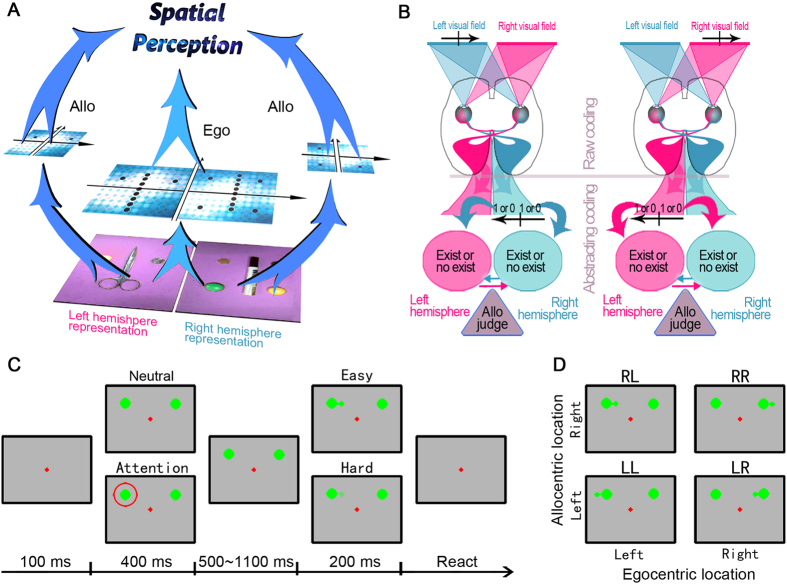
Schematic of spatial perception based on egocentric and allocentric coordinates and the allocentric position discrimination task. (**A**) Spatial encoding begins with abstracting the position information from the raw images of the visual field. Depending on the task that the subject is performing, this abstracting can be based on either egocentric (Ego) or allocentric (Allo) coordinates. Egocentric coordinates are defined as fixation-centered, and allocentric coordinates are defined as object-centered. In this figure, scissors or a whiteboard marker were defined as the center of an allocentric frame. The splits in the middle of the pictures imply that their two parts are being processed in different hemispheres. Similar to the report by Olson *et al.*, we showed that certain higher level cortical areas may code for the contralateral part of the allocentric frame, such as the SEF. The allocentric frame may be created for an instant period during the trial at hand. It remains unknown if the whole progress of allocentric spatial coding is divided into two hemispheres using this method. (**B**) The schematic shows a model of allocentric judgment with the highest level of abstraction where 1 represents existing and 0 represents not existing. First, the raw information from the visual field is projected on the contralateral V1 area. Next, the spatial encoding is abstracted from V1 by its subsequent pathways to higher-order cortical areas. As shown on the left, when the allocentric frame is defined at the left visual field, judgments in the left side of the frame (compatible condition) requires more interhemispheric cooperation than judgments in the right side of the frame (incompatible condition). The right portion is a reverse case of the allocentric frame, and it is defined in the right visual field. (**C**) The allocentric position discrimination task procedure. (**D**) According to the locations of the small target dots, trials were classified into one of two conditions (compatible or incompatible). Compatible: allocentric left and egocentric left (LL) or allocentric right and egocentric right (RR); incompatible: allocentric left and egocentric right (LR) or allocentric right and egocentric left (RL).

The behavioral task began with a red fixation point appearing on the center of a gray screen (RGB color coding: 160, 160, 160; 51.6 cd/m^2^). The subjects were instructed to focus on the fixation point at all times during the trial. Next, two large green dots appeared on the screen 8° to the left or the right horizontally and 7° upwards vertically from the fixation point. On the attentional task, one of the dots appeared with a stimulus cue consisting of a red circle that was 7° in diameter. The attentional task and the neutral task were presented randomly within a block. The cue lasted for 400 ms and was followed by a randomized period of 500–1100 ms. Next, a small green dot and a short connecting line appeared on the left or right side of one of the two large green dots during the neutral task or of the cued large green dot on the attentional task. All the large green dots in our experiment had the same color and luminance (RGB color coding: 0, 255, 0; 99.8 cd/m^2^). The colors of the small dots and connecting lines differed between the easy task (RGB color coding: 0, 255, 0; 99.8 cd/m^2^) and the hard task (RGB color coding: 160, 180, 160; 61.3 cd/m^2^). The width of the connecting line was 0.1°. The diameters of the large and small dots were 1.5° and 0.6°, respectively. Once the small green dot appeared, the subjects provided a judgment as rapidly as possible using either hand to press one of two keys separated by 15 cm on the keyboard. If the subjects could not hold their focus within a 3° diameter fixation window before the onset of the small dot, the trial was terminated and restarted immediately. Trials where the subject broke fixation were not included in our data analyses. The judgment of the subjects depended on the position of the small dot relative to the large one, i.e., an allocentric position discrimination task (Fig. 1C). Because the allocentric position of the target dot could be compatible or incompatible with its egocentric position, each trial was classified into one of two conditions: the compatible condition that consisted of allocentric left and egocentric left (LL) or allocentric right and egocentric right (RR); and the incompatible condition that consisted of allocentric left and egocentric right (LR) or allocentric right and egocentric left (RL) (Fig. 1D). The RT was calculated from the onset of the small green dot until the key was pressed by the subject. If a response to a trial was faster than 200 ms or slower than 800 ms, it was considered as an abnormal reaction trial and was not used in further analysis. To encourage subjects to respond as soon as possible, a reminder would appear on the screen when the subject did not respond within 800 ms.

### Data analyses

The subjects performed 25,688 trials in total (trials where the subject broke fixation were not included), and 24,959 trials (97.16% of the total trials) were correct. Only the RTs from correct trials were recorded. The outlier trials consisted of an RT that differed by >3 SDs from the mean and were excluded. A total of 24,732 trials (98.58% of the correct trials) were used in subsequent analyses. Because of the intrinsic difference in the RT between the dominant and non-dominant hands^29^, we used adjusted RT data in subsequent analyses. The intrinsic RT difference between the two hands of each subject was calculated by subtracting the mean RT of the left hand from that of the right hand under the compatible condition. Next, the intrinsic RT difference was used to adjust the RTs by subtracting it from the RT of the left hand^11^.

We tested the variation in RT resulting from attention and task difficulty using a repeated-measures ANOVA. The influence of compatibility between allocentric and egocentric representation on RT was tested using a multi-way ANOVA.

To quantify the influence of egocentric representation on allocentric judgment, we calculated a compatibility difference index (CDI):


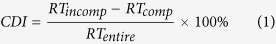


Here, RT_incomp_ and RT_comp_ represent the mean RT of incompatible and compatible trials, respectively, and RT_entire_ represents the mean RT of the entire trial set.

According to previous evidence, the two hemispheres might be asymmetric in solving conflict spatial information, i.e., the RT on the incompatible trial in the left visual hemifield is slower than the right visual hemifield^11^. To investigate the effect of different task conditions on this asymmetry, the egocentric asymmetry index (EAI) was calculated:


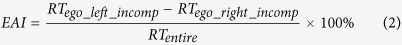


Here, RT_ego_left_incomp_ and RT_ego_right_incomp_ represent the mean RT on incompatible trials in the left and right visual hemifields, respectively.

The influence of attention and task difficulty on the CDI and EAI was examined using a paired-sample t-test. To test whether attention can reduce the CDI or the EAI to zero, a one-sample t-test was used, and a 95% confidence interval (CI) was calculated. In addition, a two-way ANOVA was used to evaluate the effects of attention and task difficulty on the CDI and EAI. The performance accuracy was not used for calculating CDI or EAI because there was no significant accuracy difference between the compatible and incompatible conditions; furthermore, previous studies suggest that the performance accuracy may be too high to be as sensitive as RT for these tasks^10^,^11^.

The handedness and dominant eye of the subjects may influence their CDI and EAI results. The effect of the Edinburgh Handedness Inventory score on the CDI and the EAI was tested using a correlation analysis, and the effect of the dominant eye on these parameters was examined using an independent t-test.

## Results

In an allocentric position judgment task, there was no significant difference in the accuracy of judgment between compatible (average accuracy of judgment: 97.16%) and incompatible (98.47%) conditions (ANOVA test, F_1,11_ = 2.11, P = 0.161) and among the four locations of the target dots (F_3,11_ = 1.59, P = 0.207; RL: 96.76%, RR: 98.61%, LL: 98.34%, LR: 97.56%). These results may be due to a performance accuracy that was too high and not as sensitive as the RT for these tasks^10^,^11^.

**Figure 2:**
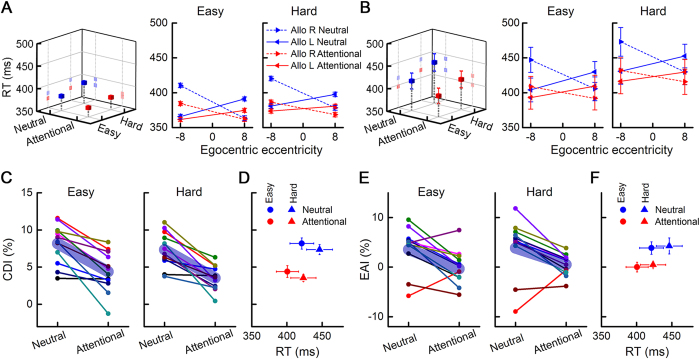
Changes in reaction time (RT), compatibility difference index (CDI) and egocentric asymmetry index (EAI) in different tasks. (**A**) RT from a representative subject. (**B**) RT from the population data. (**C**) The modulatory effects of attention and task difficulty on the CDI. The lines with the same color are from the same individual. (**D**) The relationship between RT and the CDI. (**E**) The modulatory effects of attention and task difficulty on the EAI. (**F**) The relationship between RT and the EAI.

RTs were significantly affected by both attention (Fig. 2A,B and Supplementary Figure S1A,B, repeated-measures ANOVA, F_1, 33_ = 75.01, P < 0.001) and task difficulty (F_1, 33_ = 91.55, P < 0.001). There was no significant interaction between attention and task difficulty (F_1, 33_ = 0.26, P = 0.612).

Incompatible condition trials (RL or LR in Fig. 1D) were completed significantly slower than compatible condition trials (LL or RR in Fig. 1D) in easy-neutral, easy-attentional, hard-neutral and hard-attentional tasks (Fig. 2A,B and Supplementary Figure S1A,B, multi-way ANOVA, F_1, 81_ = 135.2, P < 0.001 for compatibility effect). In addition, the CDI was significantly different between neutral and attentional tasks for the easy trials (Fig. 2C, paired-samples t-test, t_11_ = 5.29 P < 0.001) and the hard trials (t_11_ = 5.68, P < 0.001). Thus, the RT difference between compatible and incompatible conditions was significantly reduced on the attentional task compared with the neutral task.

The influence of egocentric representation on allocentric perception was asymmetric between the two visual hemifields on the neutral task according to a one-sample t-test of the EAI (Fig. 2E, t_23_ = 4.05, P < 0.001, 95% CI: 1.99–6.13). The EAI was significantly reduced by attentional modulation in both easy (paired-samples t-test, t_11_ = 3.11, P = 0.010) and hard trials (t_11_ = 2.84, P = 0.016). Moreover, the EAI was reduced to zero on the attentional task (one-sample t-test, t_23_ = 0.43, P = 0.674, 95% CI: −0.93–1.42), but the CDI was not reduced to zero (Fig. 2C, t_23_ = 15.47, P < 0.001, 95% CI: 6.73–8.81 for the neutral task; t_23_ = 8.70, P < 0.001, 95% CI: 3.03–4.93 for the attentional task).

When considering the results of both the easy and hard trials, the RT was not a direct cause of the decline in the CDI or EAI (Fig. 2D,F). On the attentional task, task difficulty significantly modulated the RT (Fig. 2A,B). However, task difficulty did not modulate the CDI on the neutral task (paired-sample t, t_11_ = 1.51, P = 0.161) or the attentional task (t_11_ = 1.31, P = 0.218) or the EAI on the neutral task (t_11_ = 0.67, P = 0.515) or the attentional task (t_11_ = 0.66, P = 0.524). Thus, attention rather than task difficulty predominantly modulated the CDI and EAI.

**Figure 3:**
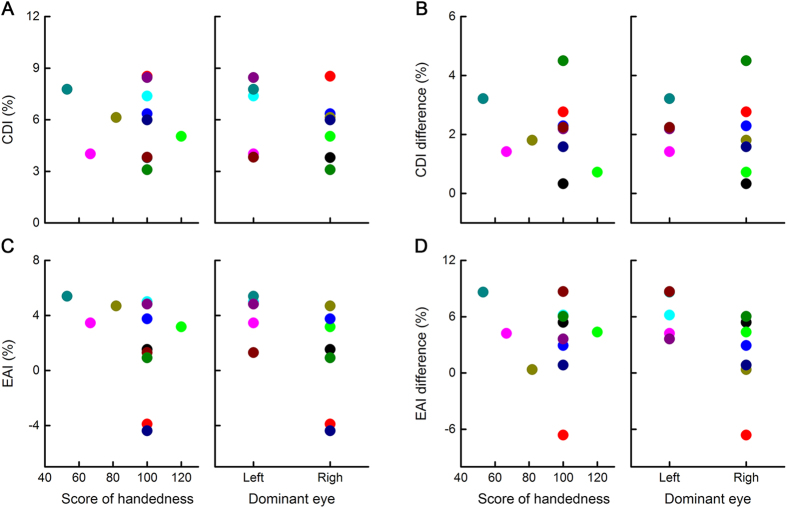
The influence of handedness and eye dominance. (**A–D**) The CDI, EAI and their fluctuations caused by attentional modulation were not influenced by handedness or eye dominance. The ordinates of (**B**,**D**) are the differences in the CDI and EAI between the neutral and attentional tasks, respectively.

To investigate whether left-right position judgments were affected by handedness or eye dominance, we recorded the handedness scores and dominant eyes of all the subjects using the *Edinburgh Handedness Inventory*^28^ and the Miles test, respectively. Correlation analysis confirmed that the handedness score did not significantly influence the CDI (Fig. 3, r = 0.13, P = 0.686), the EAI (r = 0.34, P = 0.277), or the difference in the CDI (r = 0.24, P = 0.460) or EAI (r = 0.21, P = 0.510) between the neutral and attentional tasks. Furthermore, there was no significant effect of eye dominance on the CDI (independent t-test, t_10_ = 0.63, P = 0.541), the EAI (t_10_ = 1.80, P = 0.102), or the difference in the CDI (t_10_ = 0.38, P = 0.711) or EAI between the neutral and attentional tasks (t_10_ = 2.03, P = 0.070). Here, the difference in the CDI and EAI between the neutral and attentional tasks represents the modulatory effect of attention on the CDI and EAI, respectively.

## Discussion

Both attention and task difficulty influenced the RT on the allocentric position discrimination task (Fig. 2A,B and Supplementary Figure S1A,B)^30^, but only attention modulated the characteristics of allocentric perception. This finding implies that the changes in RT caused by cue-attention and contrast-difficulty depend on different mechanisms related to allocentric location judgment; furthermore, attention, but not a reduction of difficulty, can increase the processing of conflicting information. The different RTs between compatible and incompatible tasks may be due to the influence of egocentric representation on allocentric judgment^11^ or to the influence of conflict information, and this effect was reduced by attentional modulation (Fig. 2). The reduced CDI implies that attention facilitates the conversion between egocentric and allocentric coordinates by enhancing interhemispheric cooperation^5^,^11^ or by increasing the efficacy of brain areas relevant to solving conflicting information, such as the ACC^19^.

On the neutral task, the egocentric influence on allocentric judgment was asymmetric between the two visual hemifields. According to the perspective of conflict or the flanker effect, this result implies that regions involved in resolving conflicting information, such as the ACC, process information slower in the right hemisphere than in the left during allocentric spatial perception. On the attentional task, this asymmetry was not significant. Attention may strengthen the ability of these regions in the right hemisphere to resolve conflicting information at a rate similar to the left hemisphere. Based on the asymmetric cooperation between the two hemispheres, the allocentric-egocentric incompatible condition in the left visual field requires the right hemisphere to assist the left hemisphere and vice versa (Fig. 1B). Our results suggest that the former cooperation was less efficient than the latter on the neutral task. Previous studies showed that the right hemisphere plays a more significant role in spatial perception^31^. Perplexingly, the dominance of the right hemisphere did not reduce the RT in its corresponding visual hemifield. Indeed, the RT under the incompatible condition in the left visual hemifield was slower than that of the right visual hemifield on the neutral task (Fig. 2A,B and Supplementary Figure S1A,B). Thus, the dominance of the right hemisphere in spatial perception may be due to its global effects and the reception of more information from the left hemisphere. Furthermore, the dominant hemisphere in spatial perception might be reluctant to assist the subordinate one, whereas the subordinate hemisphere might more readily assist in the neutral task. The physiological foundation of this phenomenon may be the rightward asymmetric interflow of information between the two hemispheres^11^,^32^. Based on our results, spatial attention can abolish this imbalance and enhance the communication from the right to left hemisphere.

Our results did not show a significant influence from handedness scores on the CDI and EAI. Handedness is strongly correlated with asymmetry of the vestibular cortex^33^. This indicates that the asymmetry of the vestibular cortex or other regions relevant to handedness do not have a strong correlation with asymmetric conflicting information processing. Effects of eye dominance on the CDI and EAI were not significant. However, the right eye dominant subjects had a smaller EAI value, and attention further reduced the EAI value with a moderately small statistically significant P value. Eye dominance results from visual areas across both hemispheres that prefer information from one eye over the other^34^ and partially reflects the properties of the visual cortex, which forms the basis of spatial perception. Hence, there may be a moderate correlation between eye dominance and asymmetry of egocentric-allocentric incompatible perception. The details of these relationships still need further investigation.

**Figure 4:**
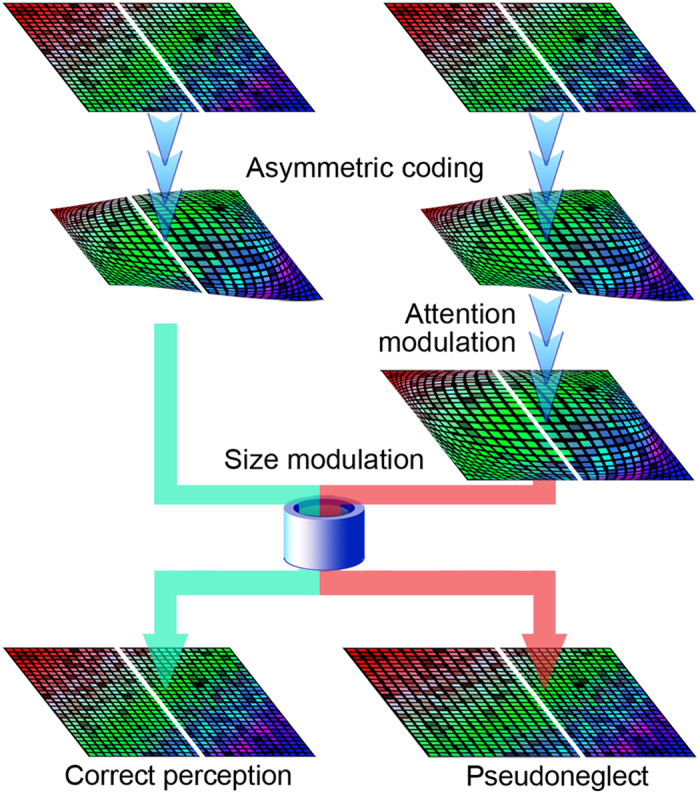
A model of interpreting pseudoneglect. The representation of visual spatial information is not evenly distributed across visual cortical areas. As shown on the left, the brain can perceive correct spatial information by resizing the coding that has developed from daily experience. As shown on the right, double resizing causes subjects to perceive that the middle of a feature deviates to the right of its actual position, i.e., pseudoneglect, when the level of attention increases.

Pseudoneglect, represented by the tendency of a normal human to bisect a line towards the left-hand side of the true center^35^, is thought to be associated with hemispheric asymmetry^36^. There is currently no consensus on the mechanism underlying pseudoneglect. Based on the current results, we suggest that pseudoneglect results from a conflict between asymmetric attentional modulation and asymmetric size modulation (Fig. 4). The brain receives spatial encoding from the visual field that is not evenly distributed. Our results suggest that this information may have a greater resolution on the right side than on the left side and centrally than on the periphery. Nevertheless, people do not perceive the visual field as imbalanced due to a brain function that resizes spatial encoding. This size modulation may be calibrated or developed from daily experience. For example, young owls can adjust auditory and visual location errors induced by experimental interventions^37^. Our results show that attention modulates asymmetric spatial encoding during spatial perception. When the level of attention is higher than in the previous experience of size modulation development, the two modulations with unique directions doubly resize the perception. As a result, an individual perceives the middle of an object as more to the left than its actual position. Attention combined with sufficient time to exert an effect caused pseudoneglect to appear in studies that provided subjects with more time to bisect lines on paper^35^. However, when the display time of the objects was limited, e.g., presenting the lines for only 100 ms^10^, pseudoneglect was not apparent.

Attentional modulation on information processing may not act as a zoom lens or spotlight^23^. However, this modulation may involve optimizing the current cooperation between the hemispheres for the work at hand. Importantly, many other cerebral functions that are asymmetric between hemispheres^1^, such as learning, memory and thinking, might be voluntarily modulated. If so, it may be possible to exploit the non-dominant hemisphere to enhance a particular behavioral performance using the appropriate training strategies.

## Additional Information

**How to cite this article**: Wang, M. *et al.* Visual attention modulates the asymmetric influence of each cerebral hemisphere on spatial perception. *Sci. Rep.* **6**, 19190; doi: 10.1038/srep19190 (2016).

## Supplementary Material

Supplementary Information
