# Supplementary material for: Visual attention modulates the asymmetric influence of each cerebral hemisphere on spatial perception
Source: Sci Rep. 2016 Jan 13;6:19190. doi: 10.1038/srep19190 (PMC4725350; doi:10.1038/srep19190)
Supplement: Supplementary Information [file srep19190-s1.pdf]

Visual attention modulates the asymmetric influence of each cerebral hemisphere on spatial perception

Meijian Wang[1], Xiuhai Wang[2], Lingyan Xue[1,3], Dan Huang[1], Yao Chen[1]*

Affiliations:

[1]School of Biomedical Engineering, Shanghai Jiao Tong University, Shanghai, 200240, China

[2]Medical College, Qingdao University, Qingdao, 266021, China

[3]School of Quality and Technical Supervision, Hebei University, Baoding, 071002, China

*Corresponding author. E-mail: yao.chen@sjtu.edu.cn

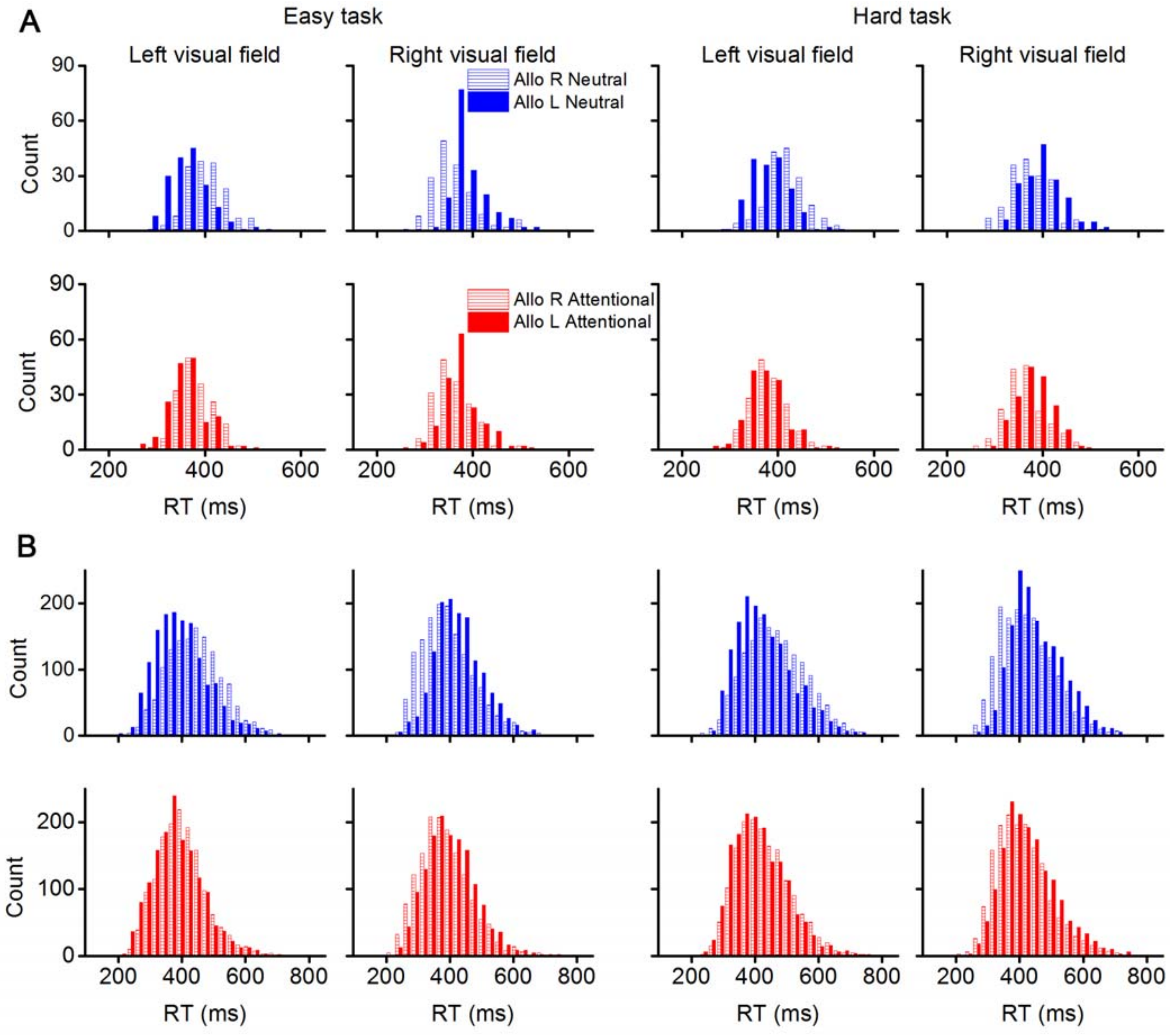

Supplementary Figure S1| Reaction times to each condition. A, RTs from a same example subject as Figure 1A. **B,** RTs from population data.
